# Research progress in abnormal carbohydrate, lipid, and protein metabolism in children with isolated growth hormone deficiency

**DOI:** 10.3389/fendo.2025.1553008

**Published:** 2025-05-22

**Authors:** Tingting Zhao, Yan Ma, Zhimin Zheng, Panpan Zhang, Hao Sun

**Affiliations:** ^1^ Dalian Women and Children’s Medical Center (Group), Dalian, Liaoning, China; ^2^ Graduate School of Dalian Medical University, Dalian, Liaoning, China

**Keywords:** growth hormone deficiency, child, growth hormone therapy, metabolism, rHGH

## Abstract

**Introduction:**

Growth hormone deficiency (GHD) is a rare endocrine disorder characterized by reduced or insufficient secretion of growth hormone (GH). GHD manifests with a heterogenous spectrum of symptoms mainly affecting musculoskeletal and endocrine system, with an increased predisposition to psychosocial disorders and ultimately reduced quality of life.

**Method:**

We searched the main databases for studies describing the metabolic effects of growth hormone and its deficiency. All full-text articles and major reviews were manually searched for additional studies.

**Result:**

Metabolic derangements of GHD are reported in carbohydrate (insulin resistance, diabetes mellitus), lipid (hypercholesterolemia, hypertriglyceridemia), and protein (decreased lean body mass) metabolism. Currently, recombinant growth hormone (rhGH) replacement therapy is the primary standard of care for the treatment of GHD. Understanding the impact of GH, including the effect of GHD and rhGH therapy, on metabolism would be a critical step in development of effective treatment and selecting the best management strategies.

**Discussion:**

This article reviews research progress and aims to offer new perspectives on the metabolic state of patients suffering from GHD and to compile data about the impact of rhGH therapy on metabolic pathways.

## 1 Introduction

Growth hormone (GH), a vital peptide hormone synthesized and secreted by cells of the anterior pituitary gland, plays a central role in promoting children’s growth and development (1). Growth hormone deficiency (GHD) is a rare endocrine disorder characterized by reduced or insufficient secretion of GH, leading to growth and developmental impairments in children. The prevalence of GHD ranges from 1:4000 to 1:10000 (2).

GHD manifests with a heterogenous spectrum of symptoms affecting musculoskeletal system (growth failure, decreased muscle tone, osteoporosis), endocrine system (delayed puberty, obesity), with an increased predisposition to psychosocial disorders (impaired sleep, anxiety, depression, low self-esteem and social isolation) (3–6). Metabolic derangements of GHD are reported in carbohydrate (insulin resistance, diabetes mellitus), lipid (hypercholesterolemia, hypertriglyceridemia), and protein (decreased lean body mass) metabolism, a clinical profile similar to Metabolic syndrome (7). Furthermore, fasting hypoglycemia is a common metabolic characteristic of infants and children with GH secretion defects. These patients exhibit increased insulin sensitivity, which enhances glucose utilization and results in fasting hypoglycemia. The hypoglycemia may result from reduced gluconeogenesis, abnormal glycogenolysis, or a combination of both, leading to reduced liver glucose output. In severe cases, repeated hypoglycemic episodes may even cause brain damage (8).

GHD is categorized into isolated form or in association with other pituitary hormone deficiencies. Most patients suffer from isolated GHD which is often idiopathic (9). For the sake of simplicity, when we refer to GHD in the rest of the paper, we mean the isolated form. GHD may be further sub-classified in etiology as congenital or acquired (due to tumor, trauma, inflammation/infection, or radiotherapy) (10).

The current available treatment for GHD is growth hormone substitution as early as diagnosis is established. For this purpose, the recombinant growth hormone (somatropin or its analogues) is administered subcutaneously with short-term (once-daily) or long-term (once-weekly) formulations (11). Patients with congenital GHD need recombinant human GH replacement therapy at an early stage (often after first year of life) to promote normal growth and development. Patients with acquired GHD may also require additional targeted treatment based on the underlying cause, including surgical treatment of brain lesions (5, 12, 13).

Although the major purpose of treatment in GHD is focused on improving stature and body gesture, GH is shown to also improve metabolism (14). GH increases amino acid uptake and protein synthesis and at the same time reduces the protein catabolism (15). In addition, GH enhances lipolysis, by promoting breakdown of triglycerides in adipose tissue, and alters distribution of lipids, often leading to a decrease in visceral and an increase in subcutaneous fat (16, 17). Such contributions to body composition are evidenced by improved lean body mass, reduced fat mass, and ultimately quality of life following GH replacement therapy (14). Understanding the impact of GH on metabolism, including the metabolic consequences of GH deficiency and GH replacement therapy, would be a critical step in development of effective treatment and selecting the best management strategies.

In recent years, the impact of growth hormone deficiency on carbohydrate, lipid, and protein metabolism in children has become a prominent area of research. This article reviews the research progress in this area to provide new ideas for the diagnosis and treatment of GHD.

## 2 GH and glucose metabolism

### 2.1 Effects of GH and GHD on glucose metabolism

**Figure 1:**
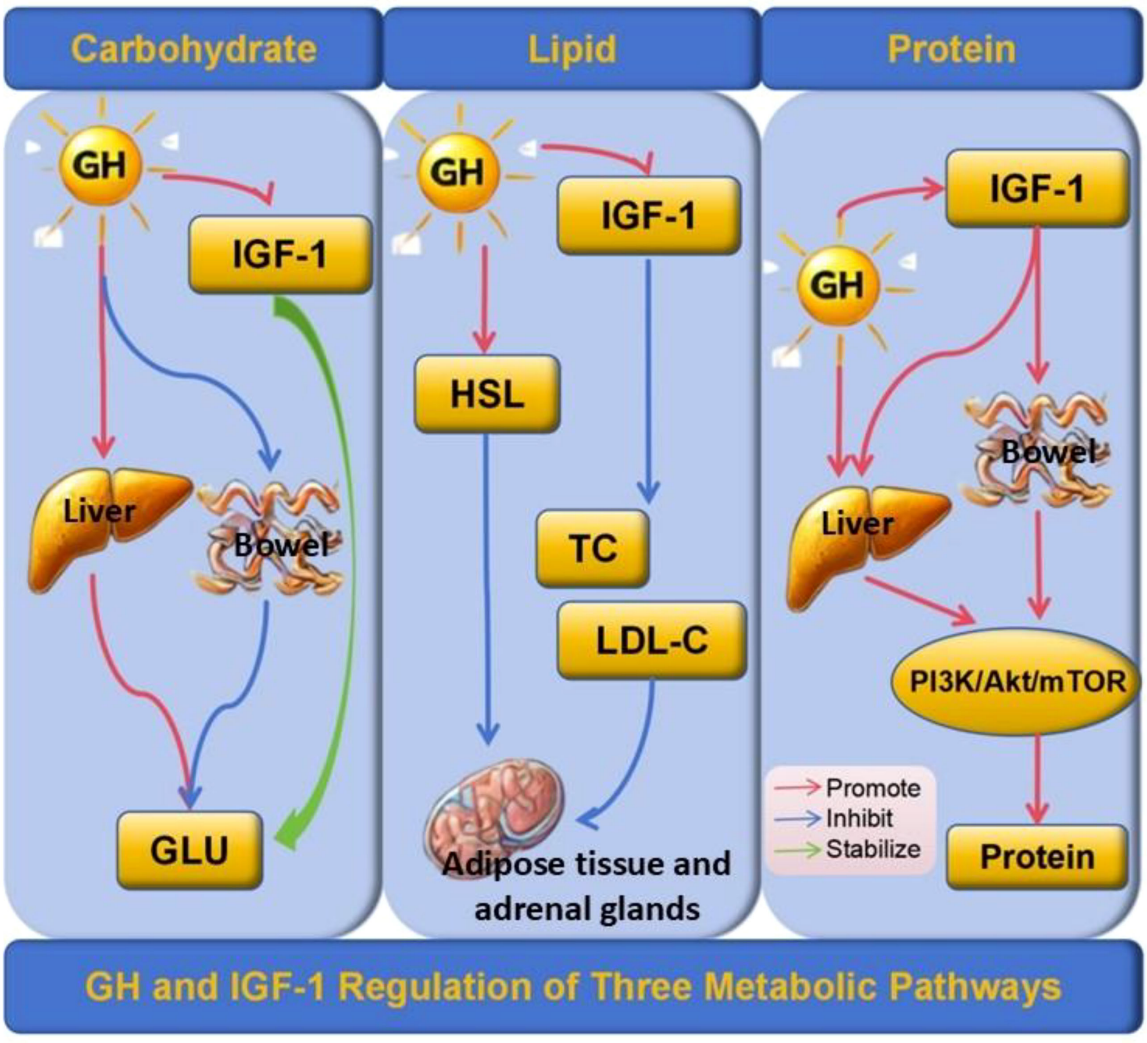
GH and IGF-1 Regulation of Three Metabolic Pathways. GH inhibits glucose uptake in peripheral tissues and promotes hepatic gluconeogenesis and glycogenolysis, while IGF-1 stabilizes blood glucose levels by enhancing glucose utilization and insulin sensitivity. In lipid metabolism, GH activates hormone-sensitive lipase (HSL), promoting triglyceride breakdown in adipose tissue and steroidogenesis in adrenal glands, and reducing total cholesterol (TC) and low-density lipoprotein cholesterol (LDL-C) levels, with IGF-1 complementing these effects by improving lipid profiles. In protein metabolism, GH and IGF-1 enhance protein synthesis through the PI3K/Akt/mTOR signaling pathway, with GH also increasing amino acid uptake and reducing protein degradation, thereby stabilizing protein metabolism. GH, Growth Hormone; IGF-1, Insulin-like Growth Factor 1; HSL, Hormone-Sensitive Lipase; TC, Total Cholesterol; LDL-C, Low-Density Lipoprotein Cholesterol; GLU, Glucose; PI3K/Akt/mTOR, Phosphoinositide 3-Kinase/Protein Kinase B/Mechanistic Target of Rapamycin. Arrows: Red, Promote; Blue, Inhibit; Green, Stabilize.

GH can interact with insulin to maintain blood sugar stability. GH can also directly inhibit glucose utilization by muscle and adipose tissue, promote hepatic gluconeogenesis and glycogenolysis, and thereby regulate blood sugar levels (16, 18, 19). GH also modulate the glucose hemostasis through leptin-receptor (LepR) expressing cells in brain, which is supported by the evidence of impaired hepatic insulin sensitivity in GHR-ablated LepR expressing neurons (8, 20). Of note, several pathways in GH signaling, including GHR/JAK2/SHC/MAPK, GHR/JAK2/STATs, and IRS/PI3K/Akt pathways, are shared with insulin, which further explains the fundamentals for metabolic interaction between GH and insulin (21) (**Figure 1**).

Several studies on mice and human with GH defect have elaborated on the role of GH in glucose metabolism and its clinical consequences. Dominici et al. reported that mice with GHD and GHR deficiency exhibited hypoglycemia, low insulin levels, and enhanced insulin sensitivity (22). Yakar et al. found that transgenic mice lacking GHR and overexpressing GH antibodies also displayed high insulin sensitivity and reduced blood glucose and blood insulin levels (23). The effect of GHD on glucose metabolism in the clinical setting is further supported by Xu et al. using metabolomics. By using NMR-based serum metabolomics, they compared 35 healthy children with 45 children with GHD-induced short stature. The study found that the serum levels of α-glucose, β-glucose, serine, lysine, and glutamine were significantly reduced in children with GHD, while the serum levels of citric acid, phenylalanine, and tyrosine were significantly increased. After conducting a comprehensive metabolic pathway analysis of the differential metabolites, Xu et al. concluded that the observed changes resulting from GH deficiency inhibit gluconeogenesis, glycolysis, and the conversion of carbohydrates into amino acids (18). Similarly, Lan Li et al. conducted fecal metabolomics analysis on short children with different GH levels and discovered that glycolysis, gluconeogenesis, and pyruvate metabolism are some key metabolic pathways that differ between children with GHD, children with idiopathic short stature (ISS), and healthy children (24).

### 2.2 Effects of rhGH replacement therapy on glucose metabolism

GH and its analogues are important drivers of the glucose metabolism and may induce hyperglycemia (25). Some studies suggest long-term rhGH replacement therapy is associated with increased level of insulin and impaired insulin sensitivity (26, 27) (**Table 1**). Meazza et al. compared 16 children with GHD undergoing rhGH replacement therapy with 20 healthy children. After 12 months of rhGH replacement therapy, they observed no significant changes in serum glucose levels. However, there was an increase in insulin levels, accompanied by decreased insulin sensitivity (28). This result was also supported by Wang et al., who treated 17 children with GHD for 3 to 30 months using rhGH and found no significant changes in blood sugar levels, but observed an increase in insulin levels (33). Saenger also reported slightly elevated insulin levels and normal blood glucose levels after 36 months of rhGH treatment in a group of children with GHD (34). Additionally, a large sample of long-term follow-up clinical data involving 100 children with GHD who underwent rhGH replacement therapy for 5 years displayed no significant changes in blood sugar and only slightly impaired insulin sensitivity (35).

**Table 1:** Effects of rhGH replacement therapy on metabolism.

Metabolic Type	Post-Treatment Improvement	Indicator Changes	Influencing Factors	References
Carbohydrate	Partial restoration of glucose levels, slight decrease in insulin sensitivity	Increased insulin levels, mild rise or stable glucose levels; minor insulin resistance in some patients	Hormone dosage, treatment duration, follow-up time, individual variability	(28–30)
Lipid	Improvement in lipid abnormalities, reduced cardiovascular risk	Significant reduction in TC and LDL-C; slight increase or stable HDL-C; increased adiponectin levels	Baseline metabolic status, treatment frequency, age	(17, 30)
Protein	Enhanced amino acid utilization, improved protein synthesis	Marked increase in IGF-1 levels, elevated amino acids (e.g., lysine, valine), reduced urea production	IGF-1 sensitivity, baseline metabolic state	(31, 32)

TC, total cholesterol; LDL-C, low-density lipoprotein; HDL-C, high-density lipoprotein; IGF-1, Insulin-like Growth Factor 1.

Some studies have reported that the fasting blood glucose levels of children with GHD can also be significantly increased during rhGH replacement therapy (29, 36, 37). Giordano et al. studied 51 children with GHD undergoing rhGH replacement therapy and found varying degrees of increase in fasting blood glucose levels after therapy (37). Likewise, Xue et al. also noted a significant increase in fasting blood glucose during 6 months of rhGH replacement therapy in 60 children, with half of the participants receiving high doses of rhGH (0.1 U/kg) (36). Ciresi et al. examined the effect of two different weekly injection schedules on the metabolic outcomes in children with GHD. The results revealed that both thrice-weekly and daily rhGH replacement injections increased fasting blood sugar in children, but rhGH replacement therapy injected three times a week had a more pronounced effect on blood sugar elevation (29).

Exogenous supplementation of rhGH affects insulin levels and sensitivity, promotes gluconeogenesis and glycogenolysis, and decreases glucose uptake in muscle and adipose tissue (15). An *in vivo* study in mice showed a GH-induced increase in expression of two pivotal genes in gluconeogenesis including phosphoenolpyruvate carboxy-kinase and glucose-6-phosphatase (38). In addition, another study on rats expressing human GH found increased activity of the glycogen synthase (39). *In vitro* studies showed that GH upregulates the p85 subunit in the phosphoinositide 3-kinase (PI3K) pathway, which decreases the PI3K, a mediator downstream of the insulin receptor, further explaining the insulin insensitivity in rhGH replacement therapy (40). In clinical setting, variations in study outcomes may be due to differences in hormone types, dosages, administration frequency, follow-up duration, and sample sizes (41, 42), highlighting the need for further research to understand the complex molecular mechanisms behind abnormal glucose metabolism in pediatric GHD.

## 3 GH and lipid metabolism

### 3.1 Effects of GH and GHD on body lipid metabolism

GH exhibits a significant lipid-lowering effect, potentially through β-adrenergic activation, which stimulates hormone-sensitive lipase, thereby indirectly promoting fat decomposition and fatty acid oxidation. It also stimulates the expression of low-density lipoprotein receptor (LDL-R) in the liver, facilitating the clearance of low-density lipoprotein cholesterol (LDL-C), and regulating lipoprotein metabolism. Consequently, GHD can disrupt lipid homeostasis, leading to abnormal lipid profiles (17, 43, 44) (**Figure 1**).

Many studies have reported that GHD significantly impairs lipid-lowering function, leading to fat accumulation. For instance, Misra et al. reported that the body fat of children with GHD was considerably high, particularly concentrated in the waist and hips, resulting in an almost 35% increase in waist-to-hip ratio (45). Similarly, Lanes et al. revealed that children with GHD exhibited increased body fat content and adipose tissue accumulation in the epicardium, potentially serving as a significant risk factor for cardiovascular disease (46). Additionally, GHD may also alter various biochemical indicators in the body. Several studies have shown that cholesterol (TC), triglyceride (TG), and low-density lipoprotein-cholesterol (LDL-C) were significantly higher in children with GHD compared to healthy children, while their levels of high-density lipoprotein-cholesterol (HDL-C) are notably lower (17, 44).

Secreted by adipocytes, adiponectin is an endogenous bioactive polypeptide or protein. Both adiponectin and fatty acid levels are altered in children with GHD. Several studies have reported that some fatty acids are significantly lowered in children with GHD compared to healthy children (24, 47, 48). Lin Junhong et al. reported that adiponectin levels in children with GHD were significantly reduced, with adiponectin negatively correlating with TG. They speculated that GH may be directly involved in regulating blood lipids in children with GHD, with adiponectin potentially playing a partial role in TG metabolism (17). Guoyou et al. performed non-targeted metabolomic analysis on the serum of 29 children with GHD, 31 children with ISS, and 30 healthy children. They found that methyl arachidonic acid was significantly reduced in the GHD group and that lipid signaling and sphingolipid metabolism pathways were significantly enriched (47).

Another mediator of the lipid metabolism and GH is betaine, a modified amino acid consisting of glycine with three methyl groups. Betaine levels in children with GHD are shown to be significantly lower compared to healthy children (18). Betaine considerably increases serum GH and IGF-1 levels, promoting fat transport and affecting lipid metabolism. It enhancesβ-oxidation of fatty acids to acetyl coenzyme A, and provides methyl groups for the conversion of phosphatidylethanolamine to phosphatidylcholine, thereby reducing malate dehydrogenase activity (18). Reduced serum levels of betaine, along with other biomarkers including serine, glutamine, lysine, α- & β-glucose, myo-inositol, and glycerol could be used as an indicator for diagnosis of GHD. In addition, exogenous supplemental betaine and endogenous increase in lysine and glutamine could improve lipid metabolism and thus might be regarded as potential treatments of GHD (18, 49). However, there are currently limited studies on this topic, and require further clinical trials.

### 3.2 Effects of rhGH replacement therapy on body lipid metabolism

Lipid metabolism disorders have been shown to be improved by standardized rhGH replacement therapy. While research findings on the effect of rhGH replacement therapy on lipid metabolism in children with GHD vary, the general consensus is that rhGH replacement therapy can reduce blood TC and LDL-C levels, with either no impact on or a potential increase in HDL-C levels. After 12 months of rhGH replacement therapy in 60 children with GHD, Ming et al. observed significant reductions in TC, TG, and LDL-C levels, along with an increase in HDL-C levels. Particularly, these metabolic indexes were more pronounced in children receiving high-dose rhGH replacement therapy (17). Belceanu et al. studied 42 children with GHD and found significant decreases in TC and LDL-C after 12 months of rhGH replacement therapy, though HDL-C and TG levels remained almost unchanged during the monitoring period (30).

The regulation of fatty acid metabolism may represent one of the potential mechanisms by which rhGH replacement therapy improves GHD. However, there are inconsistencies in findings across different studies. Li et al. conducted serum metabolomic analysis on 57 children with GHD undergoing rhGH replacement and observed a significant increase in stearic acid, myristic acid, palmitoleic acid, dodecanoic acid, heptadecanoic acid, and oleic acid, following the treatment. The reduction of fatty acids in patients with GHD was restored to normal levels after rhGH treatment. Additionally, the study revealed that stearic acid, myristic acid, palmitoleic acid, dodecanoic acid, and heptadecanoic acid were negatively correlated with the age of the subjects, with the younger subjects demonstrating higher clinical efficacy compared to older ones (24). Based on these findings, it was speculated that initiating rhGH replacement therapy at a younger age result in better outcomes. Meanwhile, other studies have also reported that adiponectin levels increased in children with GHD after rhGH replacement therapy, potentially due to the direct effects of GH (48). The study by Lin et al. also supported this finding but suggested that the results might be influenced by the small number of children with GHD and the wide variation in serum adiponectin levels (17). Some researchers have also reported no significant changes in adipose tissue levels after rhGH replacement therapy (50).

Another important metabolic effect of rhGH is its cumulative side effect on cardiovascular morbidity and mortality. It has been suggested that GH could increase the risk of atherosclerosis directly via triggering the inflammatory process in atherogenesis (51), by increasing the risk of type 2 diabetes mellitus (52, 53), or by inducing dyslipidemia (17). The latter has been studied with its association with a biomarker of atherosclerosis, the carotid intima-media thickness (cIMT). Saygili et al. studied 71 children with isolated GHD and found a considerable increase in cIMT in GHD patients receiving rhGH compared to the control group (52). In contrary, a study on 60 Chinese children with GHD (17) reported a significant decrease in the TC, TG, LDL-C, and cIMT, as well as a significant increase in the HDL-C following one year of rhGH therapy. This result was further supported by a recent prospective study on 24 children with GHD reported improved lipid profile and cIMT after receiving one year of rhGH (54). The evidences of rhGH on lipid profile and risk of cardiovascular disorders are not univocal and this topic is still partially matter of debate. Further prospective research on the effect of rhGH on the risk of atherosclerosis in patients with GHD is required to establish a uniformed guideline on monitoring of cardiovascular system. Until then, close monitoring of the lipid metabolism in patients with GHD is highly recommended to mitigate the potential adverse effects of lipid metabolism disorders.

## 4 GH and protein metabolism

### 4.1 Effects of GH and GHD on protein metabolism

Under normal eating conditions, GH plays a significant role in protein metabolism. It not only promotes protein synthesis in muscles and at the systemic level but also enhances the utilization of amino acids for protein synthesis. GH also helps reduce protein decomposition by decreasing amino acid oxidation, amino acid degradation, and hepatic urea formation. GH also modulates the maintenance of the protein pool under fasting and stress conditions (31, 55, 56). Children with GHD experience abnormal protein metabolism, leading to specific metabolic changes in amino acids and ultimately resulting in growth restriction. Amino acids are the building blocks of proteins. Lysine is an essential amino acid, which promotes the secretion of GH receptors in tissues. The receptors bind to GH, which in turn induces IGF-1, promoting growth and development (**Figure 1**). Studies have shown that lysine levels in children with GHD are significantly lower than those in normal children, which may contribute to disorders in the growth and development of children with GHD (18, 57, 58). Moreover, some studies have reported that GHD is associated with decreased valine and phenylacetate levels, along with increased acetate levels. Notably, valine and phenylacetate levels are positively correlated with GH peak levels (24). Valine is an essential bioactive molecule that plays a crucial role in protein synthesis, energy metabolism, and nutrient absorption. Phenylacetic acid is a bacterial metabolite derived from aromatic amino acids that can disrupt redox homeostasis. Disorders in phenylacetic acid metabolism may result in increased oxidative stress in children with GHD, thereby raising the risk of cardiovascular complications. Therefore, whether supplementing specific amino acids can improve the protein metabolism levels in children with GHD remains to be supported by extensive clinical and laboratory data and will be a key focus of future research in this area.

### 4.2 Effects of rhGH replacement therapy on protein metabolism

GH is a hormone primarily responsible for promoting protein synthesis in the human body. Liver cells are vital target organs of GH. GH has been known to exert its therapeutic effects via IGF-1. The rhGH replacement therapy has been shown to stimulate the synthesis and secretion of IGF-1 and IGFBP3 in children with GHD. IGFBP3 upregulates the activity of IGF-1, which in turn inhibits protein degradation, and enhances the amino acid uptake and cell proliferation, thus improving liver function, promoting protein synthesis, reducing protein degradation, and sustaining positive nitrogen balance in children with GHD (31, 55, 56). The rhGH replacement therapy has also been shown to reduce urea excretion, urea flux, and urea production rate while enhancing protein synthesis and promoting growth and development in children with GHD. Additionally, the rhGH replacement therapy offers application prospects to improve the body’s protein metabolism. Several studies have shown that the rhGH replacement therapy has a unique impact on promoting protein metabolism in patients undergoing surgery as well as during wound healing in burn patients (59–62). The rhGH replacement therapy also has the potential to improve gastrointestinal function. Studies have shown that rhGH promotes the intestinal uptake and utilization of glutamine, in turn, promoting intestinal cellular proliferation and repair to maintain optimal intestinal mucosal barrier function (32). Also, the intermediate products of hydroxyproline metabolism, i.e., prolyl-hydroxyproline and isoleucamide-hydroxyproline have been shown to be present in significantly elevated levels during the six months of rhGH replacement therapy. Prolyl-hydroxyproline is a critical collagen degradation product that is found in human blood circulation. The results of *in vitro* experiments have identified that it affects fibroblast proliferation, regulates chondrocyte differentiation, and promotes osteoblast differentiation by enhancing Foxol expression (43). Thus, the rhGH replacement therapy has been shown to play a critical role in improving the body’s protein metabolism (**Table 1**).

## 5 Summary and outlook

GH, a key endocrine hormone is known to play a key role in promoting optimal growth and maintaining metabolic homeostasis in children. Children with GHD have been known to exhibit apparent retardation in growth and development as well as severe abnormalities in carbohydrate, lipid, and protein metabolism. These metabolic abnormalities threaten children’s physical and psychological health. The rhGH replacement therapy has been shown to effectively improve the growth curve and metabolic status in children, in turn, promoting their growth and development and improving their quality of life.

Despite the presence of several studies on abnormal carbohydrate, lipid, and protein metabolism in children with GHD, these topics still need further in-depth study and exploration. The currently available literature is mainly focused on a particular aspect of metabolism and does not systematically explain the comprehensive impact of GHD on various metabolic levels of carbohydrates, lipids, and proteins. Also, most studies focus on detecting and observing biochemical indicators; however, they lack an in-depth discussion of the underlying mechanisms. Additionally, the sample size is small and the research subjects mostly constitute specific ethnic groups, limiting the universal applicability of these results.

Therefore, a comprehensive, multi-level, and multi-dimensional approach is required to unravel the mechanisms underlying GHD. Integrating multi-omics research tools, such as proteomics, metabolomics, and genomics will enable systemic identification of these metabolic mechanisms. These mechanisms should be validated by large-scale, long-term follow-up studies and clinical data. This will help deepen our understanding of the metabolic role of GHD and provide scientific and personalized guidance for clinical treatment.

## Data Availability

The original contributions presented in the study are included in the article/supplementary material. Further inquiries can be directed to the corresponding author.

## References

[1] Mameli C Guadagni L Orso M Calcaterra V Wasniewska MG Aversa T. Epidemiology of growth hormone deficiency in children and adolescents: a systematic review. Endocrine. (2024) 85:91–8. doi: 10.1007/s12020-024-03778-4 PMC11246253 38498128

[2] Stanley T. Diagnosis of growth hormone deficiency in childhood. Curr Opin Endocrinol Diabetes Obes. (2012) 19:47–52. doi: 10.1097/MED.0b013e32834ec952 22157400 PMC3279941

[3] Brod M Alolga SL Beck JF Wilkinson L Højbjerre L Rasmussen MH. Understanding burden of illness for child growth hormone deficiency. Qual Life Res. (2017) 26:1673–86. doi: 10.1007/s11136-017-1529-1 PMC5486907 28247315

[4] Albers N Cadarette S Feakins B Arregui M Ebohon S Lai P. Long-acting growth hormone for pediatric growth hormone deficiency. J Endocr Soc. (2025) 9:bvaf040. doi: 10.1210/jendso/bvaf040 40144813 PMC11938432

[5] Boguszewski MCS. Growth hormone deficiency and replacement in children. Rev Endocr Metab Disord. (2021) 22:101–8. doi: 10.1007/s11154-020-09604-2 33029711

[6] Shafiei M Hosseini S Ghadimi S Mirzaee M Keikhah M Ardalan N. Renal disorders in Autoimmune Polyendocrinopathy Candidiasis Ectodermal dystrophy (APECED): a systematic review. BMC Pediatr. (2025) 25:139. doi: 10.1186/s12887-025-05458-2 40000975 PMC11863426

[7] Rothermel J Reinehr T. Metabolic alterations in paediatric GH deficiency. Best Pract Res Clin Endocrinol Metab. (2016) 30:757–70. doi: 10.1016/j.beem.2016.11.004 27974189

[8] Cady G Landeryou T Garratt M Kopchick JJ Qi N Garcia-Galiano D. Hypothalamic growth hormone receptor (GHR) controls hepatic glucose production in nutrient-sensing leptin receptor (LepRb) expressing neurons. Mol Metab. (2017) 6:393–405. doi: 10.1016/j.molmet.2017.03.001 28462074 PMC5404104

[9] Murray PG Clayton PE. Disorders of growth hormone in childhood. In: Feingold KR Ahmed SF Anawalt B Blackman MR Boyce A Chrousos G, editors. Endotext. South Dartmouth (MA): MDText.com, Inc. Copyright © 2000-2025, MDText.com, Inc.. (2000). 25905205

[10] Hage C Gan HW Ibba A Patti G Dattani M Loche S. Advances in differential diagnosis and management of growth hormone deficiency in children. Nat Rev Endocrinol. (2021) 17:608–24. doi: 10.1038/s41574-021-00539-5 34417587

[11] Cuboni D Aversa LS Grottoli S Ghigo E Gasco V. An overview of the controversies of adult growth hormone deficiency diagnosis. Expert Rev Endocrinol Metab. (2025) p:1–14. doi: 10.1080/17446651.2025.2480699 40160150

[12] Fideleff HL Boquete HR Suárez MG Azaretzky M. Burden of growth hormone deficiency and excess in children. Prog Mol Biol Transl Sci. (2016) 138:143–66. doi: 10.1016/bs.pmbts.2015.10.009 26940390

[13] Grimberg A DiVall SA Polychronakos C Allen DB Cohen LE Quintos JB. Guidelines for growth hormone and insulin-like growth factor-I treatment in children and adolescents: growth hormone deficiency, idiopathic short stature, and primary insulin-like growth factor-I deficiency. Horm Res Paediatr. (2016) 86:361–97. doi: 10.1159/000452150 27884013

[14] Ferruzzi A Vrech M Pietrobelli A Cavarzere P Zerman N Guzzo A. The influence of growth hormone on pediatric body composition: A systematic review. Front Endocrinol (Lausanne). (2023) 14:1093691. doi: 10.3389/fendo.2023.1093691 36843617 PMC9947344

[15] Aghili ZS Khoshnevisan G Mostoli R Alibaglouei M Zarkesh-Esfahani SH. Growth hormone signaling and clinical implications: from molecular to therapeutic perspectives. Mol Biol Rep. (2025) 52:202. doi: 10.1007/s11033-025-10304-w 39904816

[16] Sakharova AA Horowitz JF Surya S Goldenberg N Harber MP Symons K. Role of growth hormone in regulating lipolysis, proteolysis, and hepatic glucose production during fasting. J Clin Endocrinol Metab. (2008) 93:2755–9. doi: 10.1210/jc.2008-0079 PMC2453052 18413425

[17] Chen M Gan D Luo Y Rampersad S Xu L Yang S. Effect of recombinant human growth hormone therapy on blood lipid and carotid intima-media thickness in children with growth hormone deficiency. Pediatr Res. (2018) 83(5):954–60. doi: 10.1038/pr.2017.271 PMC6023698 29206809

[18] Xu R Zhu H Zhang C Shen G Feng J. Metabolomic analysis reveals metabolic characteristics of children with short stature caused by growth hormone deficiency. Clin Sci (Lond). (2019) 133:777–88. doi: 10.1042/CS20181005 30867230

[19] Brooks NL Trent CM Raetzsch CF Flurkey K Boysen G Perfetti MT. Low utilization of circulating glucose after food withdrawal in Snell dwarf mice. J Biol Chem. (2007) 282:35069–77. doi: 10.1074/jbc.M700484200 17905742

[20] Donato J Jr. Wasinski F Furigo IC Metzger M Frazão R. Central regulation of metabolism by growth hormone. Cells. (2021) 10:129. doi: 10.3390/cells10010129 33440789 PMC7827386

[21] Qiu H Yang JK Chen C. Influence of insulin on growth hormone secretion, level and growth hormone signalling. Sheng Li Xue Bao. (2017) 69:541–56. doi: 10.13294/j.aps.2017.0062 29063103

[22] Dominici FP Argentino DP Muñoz MC Miquet JG Sotelo AI Turyn D. Influence of the crosstalk between growth hormone and insulin signalling on the modulation of insulin sensitivity. Growth Horm IGF Res. (2005) 15:324–36. doi: 10.1016/j.ghir.2005.07.001 16112592

[23] Yakar S Setser J Zhao H Stannard B Haluzik M Glatt V. Inhibition of growth hormone action improves insulin sensitivity in liver IGF-1-deficient mice. J Clin Invest. (2004) 113:96–105. doi: 10.1172/JCI200417763 14702113 PMC300761

[24] Li L Wang Y Huang Y Lu Y Wang W Chen X. Impact of different growth hormone levels on gut microbiota and metabolism in short stature. Pediatr Res. (2024) 96:115–23. doi: 10.1038/s41390-024-03140-4 38582946

[25] Kim SH Park MJ. Effects of growth hormone on glucose metabolism and insulin resistance in human. Ann Pediatr Endocrinol Metab. (2017) 22:145–52. doi: 10.6065/apem.2017.22.3.145 PMC5642081 29025199

[26] Scarano E Riccio E Somma T Arianna R Romano F Di Benedetto E. Impact of long-term growth hormone replacement therapy on metabolic and cardiovascular parameters in adult growth hormone deficiency: comparison between adult and elderly patients. Front Endocrinol (Lausanne). (2021) 12:635983. doi: 10.3389/fendo.2021.635983 33716985 PMC7947790

[27] Fathallah N Slim R Larif S Hmouda H Ben Salem C. Drug-induced hyperglycaemia and diabetes. Drug Saf. (2015) 38:1153–68. doi: 10.1007/s40264-015-0339-z 26370106

[28] Meazza C Elsedfy HH Pagani S Bozzola E El Kholy M Bozzola M. Metabolic parameters and adipokine profile in growth hormone deficient (GHD) children before and after 12-month GH treatment. Horm Metab Res. (2014) 46:219–23. doi: 10.1055/s-0033-1358730 24297484

[29] Ciresi A Cicciò F Radellini S Guarnotta V Calcaterra AM Giordano C. More favorable metabolic impact of three-times-weekly versus daily growth hormone treatment in naïve GH-deficient children. Int J Endocrinol. (2017) 2017:8469680. doi: 10.1155/2017/8469680 28634491 PMC5467351

[30] Belceanu AD Bîlha ŞC Vulpoi C Brănişteanu DD. The impact of growth hormone replacement therapy on adipokines, but not upon ghrelin. Minerva Endocrinol (Torino). (2023) 48:411–9. doi: 10.23736/S2724-6507.21.03588-0 34546018

[31] Møller N Jørgensen JO. Effects of growth hormone on glucose, lipid, and protein metabolism in human subjects. Endocr Rev. (2009) 30:152–77. doi: 10.1210/er.2008-0027 19240267

[32] Wales PW Nasr A de Silva N Yamada J. Human growth hormone and glutamine for patients with short bowel syndrome. Cochrane Database Syst Rev. (2010) 6:Cd006321. doi: 10.1002/14651858.CD006321.pub2 PMC12947059 20556765

[33] Wang C Huang H Zhao C Zhao J Xiong R Jin R. The impact of pegylated recombinant human growth hormone replacement therapy on glucose and lipid metabolism in children with growth hormone deficiency. Ann Palliat Med. (2021) 10:1809–14. doi: 10.21037/apm-20-871 33440978

[34] Saenger P. Metabolic consequences of growth hormone treatment in paediatric practice. Horm Res. (2000) 53 Suppl 1:60–9. doi: 10.1159/000053207 10895045

[35] Capalbo D Esposito A Improda N Wasniewska MG Di Mase R De Luca F. Glucose homeostasis in GHD children during long-term replacement therapy: a case-control study. Endocrine. (2018) 59:643–50. doi: 10.1007/s12020-017-1408-0 28875423

[36] Xue Y Gao Y Wang S Wang P. An examination of the effects of different doses of recombinant human growth hormone on children with growth hormone deficiency. Exp Ther Med. (2016) 11:1647–52. doi: 10.3892/etm.2016.3091 PMC4840766 27168784

[37] Ciresi A Giordano C. Glucose metabolism in children with growth hormone deficiency. Front Endocrinol (Lausanne). (2018) 9:321. doi: 10.3389/fendo.2018.00321 29942285 PMC6005337

[38] Kim YD Li T Ahn SW Kim DK Lee JM Hwang SL. Orphan nuclear receptor small heterodimer partner negatively regulates growth hormone-mediated induction of hepatic gluconeogenesis through inhibition of signal transducer and activator of transcription 5 (STAT5) transactivation. J Biol Chem. (2012) 287:37098–108. doi: 10.1074/jbc.M112.339887 PMC3481310 22977252

[39] Cho Y Ariga M Uchijima Y Kimura K Rho JY Furuhata Y. The novel roles of liver for compensation of insulin resistance in human growth hormone transgenic rats. Endocrinology. (2006) 147:5374–84. doi: 10.1210/en.2006-0518 16916956

[40] del Rincon JP Iida K Gaylinn BD McCurdy CE Leitner JW Barbour LA. Growth hormone regulation of p85alpha expression and phosphoinositide 3-kinase activity in adipose tissue: mechanism for growth hormone-mediated insulin resistance. Diabetes. (2007) 56:1638–46. doi: 10.2337/db06-0299 17363744

[41] Tamiji M Taheri SM Motahari SA. Stratification of admixture population: A bayesian approach. In: 2019 7th Iranian joint congress on fuzzy and intelligent systems (CFIS). Iran: Tehran University of Medical Sciences. (2019).

[42] Sapoval N Liu Z Tamiji M Li M Nakhleh L. Theoretical and empirical performance of pseudo-likelihood-based bayesian inference of species trees under the multispecies coalescent. bioRxiv. (2025). doi: 10.1101/2025.01.28.635282

[43] Y X. Characteristics of catch-up growth in small for gestational age infants and the effects and mechanisms of recombinant human growth hormone therapy on glucose and lipid metabolism. China: Peking Union Medical College (2019).

[44] Makimura H Stanley T Mun D Chen C Wei J Connelly JM. Reduced growth hormone secretion is associated with increased carotid intima-media thickness in obesity. J Clin Endocrinol Metab. (2009) 94:5131–8. doi: 10.1210/jc.2009-1295 PMC2795664 19837914

[45] Misra M Bredella MA Tsai P Mendes N Miller KK Klibanski A. Lower growth hormone and higher cortisol are associated with greater visceral adiposity, intramyocellular lipids, and insulin resistance in overweight girls. Am J Physiol Endocrinol Metab. (2008) 295:E385–92. doi: 10.1152/ajpendo.00052.2008 PMC2519763 18544645

[46] Lanes R Soros A Flores K Gunczler P Carrillo E Bandel J. Endothelial function, carotid artery intima-media thickness, epicardial adipose tissue, and left ventricular mass and function in growth hormone-deficient adolescents: apparent effects of growth hormone treatment on these parameters. J Clin Endocrinol Metab. (2005) 90:3978–82. doi: 10.1210/jc.2005-0091 15870123

[47] Chen G Wang J Jing Y Li C Zhang W Yang S. Serum metabonomics reveals key metabolites in different types of childhood short stature. Front Pharmacol. (2022) 13:818952. doi: 10.3389/fphar.2022.818952 35600884 PMC9117746

[48] Bramnert M Segerlantz M Laurila E Daugaard JR Manhem P Groop L. Growth hormone replacement therapy induces insulin resistance by activating the glucose-fatty acid cycle. J Clin Endocrinol Metab. (2003) 88:1455–63. doi: 10.1210/jc.2002-020542 12679422

[49] Zhan XA Li JX Xu ZR Zhao RQ. Effects of methionine and betaine supplementation on growth performance, carcase composition and metabolism of lipids in male broilers. Br Poult Sci. (2006) 47:576–80. doi: 10.1080/00071660600963438 17050102

[50] Salerno M Esposito V Spinelli L Di Somma C Farina V Muzzica S. Left ventricular mass and function in children with GH deficiency before and during 12 months GH replacement therapy. Clin Endocrinol (Oxf). (2004) 60:630–6. doi: 10.1111/j.1365-2265.2004.02026.x 15104568

[51] McGrath S Morris M Bouloux PM. Growth hormone deficiency and atherosclerosis–is there a link? Growth Horm IGF Res. (1999) 9 Suppl A:9–13. doi: 10.1016/s1096-6374(99)80003-3 10429874

[52] Saygılı S Kocaağa M Kaya G Şükür M Baş F Poyrazoğlu Ş. Increased carotid intima-media thickness and its association with carbohydrate metabolism and adipocytokines in children treated with recombinant growth hormone. J Clin Res Pediatr Endocrinol. (2023) 15:69–80. doi: 10.4274/jcrpe.galenos.2022.2022-8-19 36416456 PMC9976170

[53] Shajari A Zare Ahmadabadi A Ashrafi MM Mahdavi T Mirzaee M Mohkam M. Inborn errors of immunity with kidney and urinary tract disorders: a review. Int Urol Nephrol. (2024) 56:1965–72. doi: 10.1007/s11255-023-03907-4 PMC11090940 38198013

[54] Improda N Moracas C Mattace Raso G Valente V Crisci G Lorello P. Vascular function and intima-media thickness in children and adolescents with growth hormone deficiency: results from a prospective case-control study. Horm Res Paediatr. (2024) 97:140–7. doi: 10.1159/000531473 37290420

[55] Moller N Vendelbo MH Kampmann U Christensen B Madsen M Norrelund H. Growth hormone and protein metabolism. Clin Nutr. (2009) 28:597–603. doi: 10.1016/j.clnu.2009.08.015 19773097

[56] Møller N Gjedsted J Gormsen L Fuglsang J Djurhuus C. Effects of growth hormone on lipid metabolism in humans. Growth Horm IGF Res. (2003) 13 Suppl A:S18–21. doi: 10.1016/S1096-6374(03)00048-0 12914720

[57] Uauy R Kurpad A Tano-Debrah K Otoo GE Aaron GA Toride Y. Role of protein and amino acids in infant and young child nutrition: protein and amino acid needs and relationship with child growth. J Nutr Sci Vitaminol (Tokyo). (2015) 61 Suppl:S192–4. doi: 10.3177/jnsv.61.S192 26598853

[58] D’Andrea G. Classifying amino acids as gluco(glyco)genic, ketogenic, or both. Biochem Educ. (2000) 28:27–8. doi: 10.1016/S0307-4412(98)00271-4 10717451

[59] Gu GS Ren JA Li N Li JS. Effects of recombinant human growth hormone on enterocutaneous fistula patients. World J Gastroenterol. (2008) 14:6858–62. doi: 10.3748/wjg.14.6858 PMC2773883 19058314

[60] Zhang XJ Irtun O Chinkes DL Wolfe RR. Acute responses of muscle protein metabolism to reduced blood flow reflect metabolic priorities for homeostasis. Am J Physiol Endocrinol Metab. (2008) 294:E551–7. doi: 10.1152/ajpendo.00467.2007 18089763

[61] Gamrin L Essén P Hultman E McNurlan MA Garlick PJ Wernerman J. Protein-sparing effect in skeletal muscle of growth hormone treatment in critically ill patients. Ann Surg. (2000) 231:577–86. doi: 10.1097/00000658-200004000-00018 PMC1421035 10749620

[62] Armstrong PA Venugopal N Wright TJ Randolph KM Batson RD Yuen KCJ. Traumatic brain injury, abnormal growth hormone secretion, and gut dysbiosis. Best Pract Res Clin Endocrinol Metab. (2023) 37:101841. doi: 10.1016/j.beem.2023.101841 38000973

